# Supplementary material for: Capacitive neural network with neuro-transistors
Source: Nat Commun. 2018 Aug 10;9:3208. doi: 10.1038/s41467-018-05677-5 (PMC6086838; doi:10.1038/s41467-018-05677-5)
Supplement: Supplementary file 1 — Supplementary Information [file 41467_2018_5677_MOESM1_ESM.pdf]

Supplementary Information

Capacitive neural network with neuro-transistors

Wang et al.

Supplementary Figures 1 – 12;

Supplementary Note 1.

Supplementary Figures

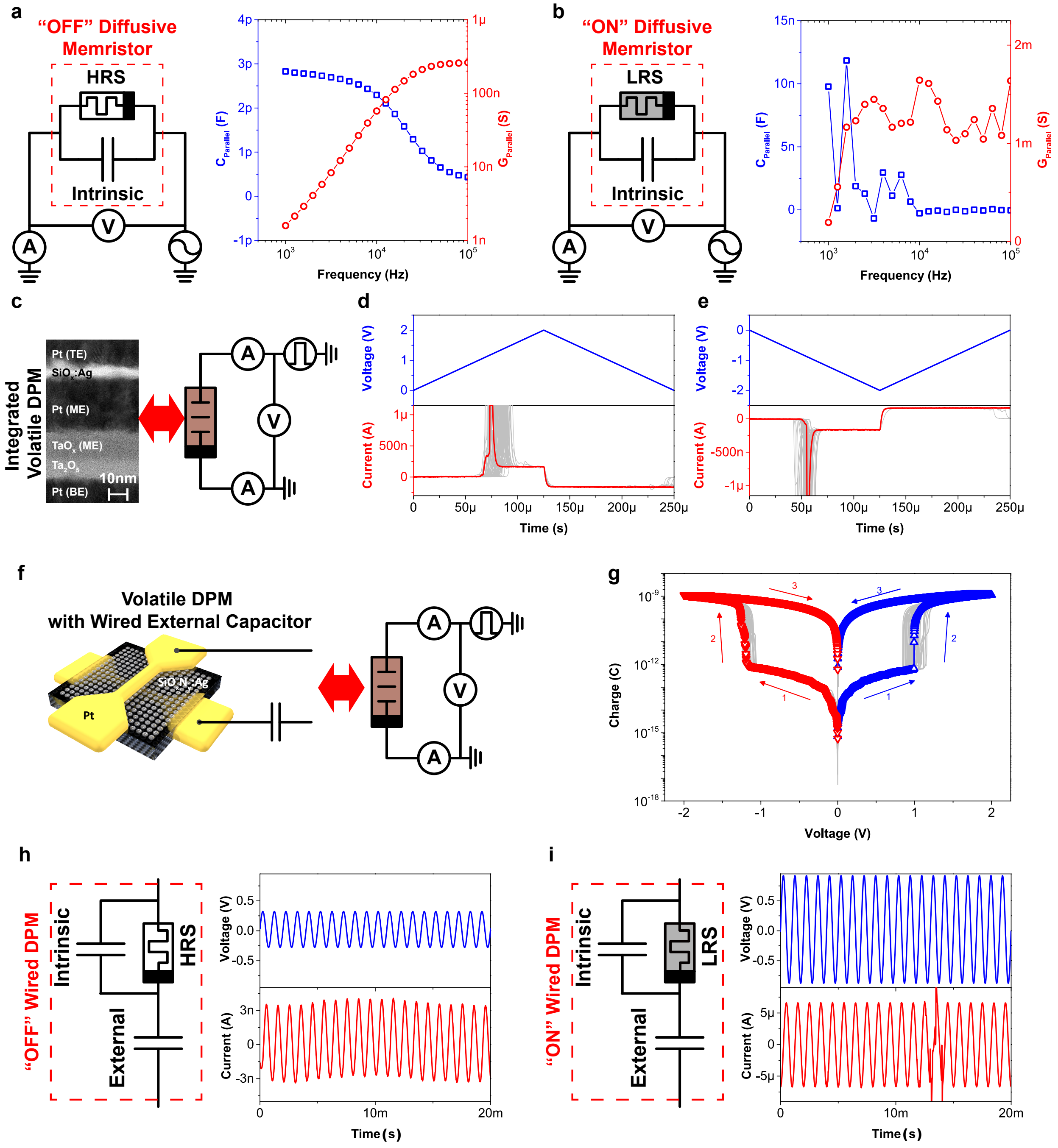

Supplementary Figure 1. Impedance of the dynamic pseudo-memcapacitor (DPM). a-b, Schematic of equivalent circuits, capacitance (C_{parallel}) - frequency responses (blue curve), and conductance (G_{parallel}) - frequency response (red curve) in the range from 1kHz to 100kHz of a diffusive memristor in its high resistance state (HRS) and low resistance state (LRS), respectively.

c, Schematics of the measurement setup used in Fig. 1b. The integrated DPM was probed by two Waveform Generator/Fast Measurement Units(WGFMUs) of Keysight B1530. WGFMU1 applied the triangular voltage waveform and measured the current. WGFMU2 stayed on the ground potential and measured the current with a smaller range and a higher resolution. The voltage was further verified by an oscilloscope. The charge was calculated by integrating the current measured over time. **d-e**, The measured current-time responses used in computing the charge hysteresis loops on both positive and negative bias in Fig. 1b. The diffusive memristor mostly relaxed after the voltage returned to zero, implying a close to 0 holding voltage. **f**, Schematics of the measurement setup for a DPM built by wiring a diffusive memristor with an off-the-shelf capacitor (1nF). **g**, Charge-voltage response of the wired DPM in **f**. **h-i**, Equivalent circuits and temporal current-voltage responses of the low capacitance state and high capacitance state of a DPM in **f**, respectively. The amplitude of the sinusoidal current observed is ~3nA(~5μA) with a ~90 degrees phase difference from the input voltage waveforms, indicating ~2.5pF(1nF) capacitance.

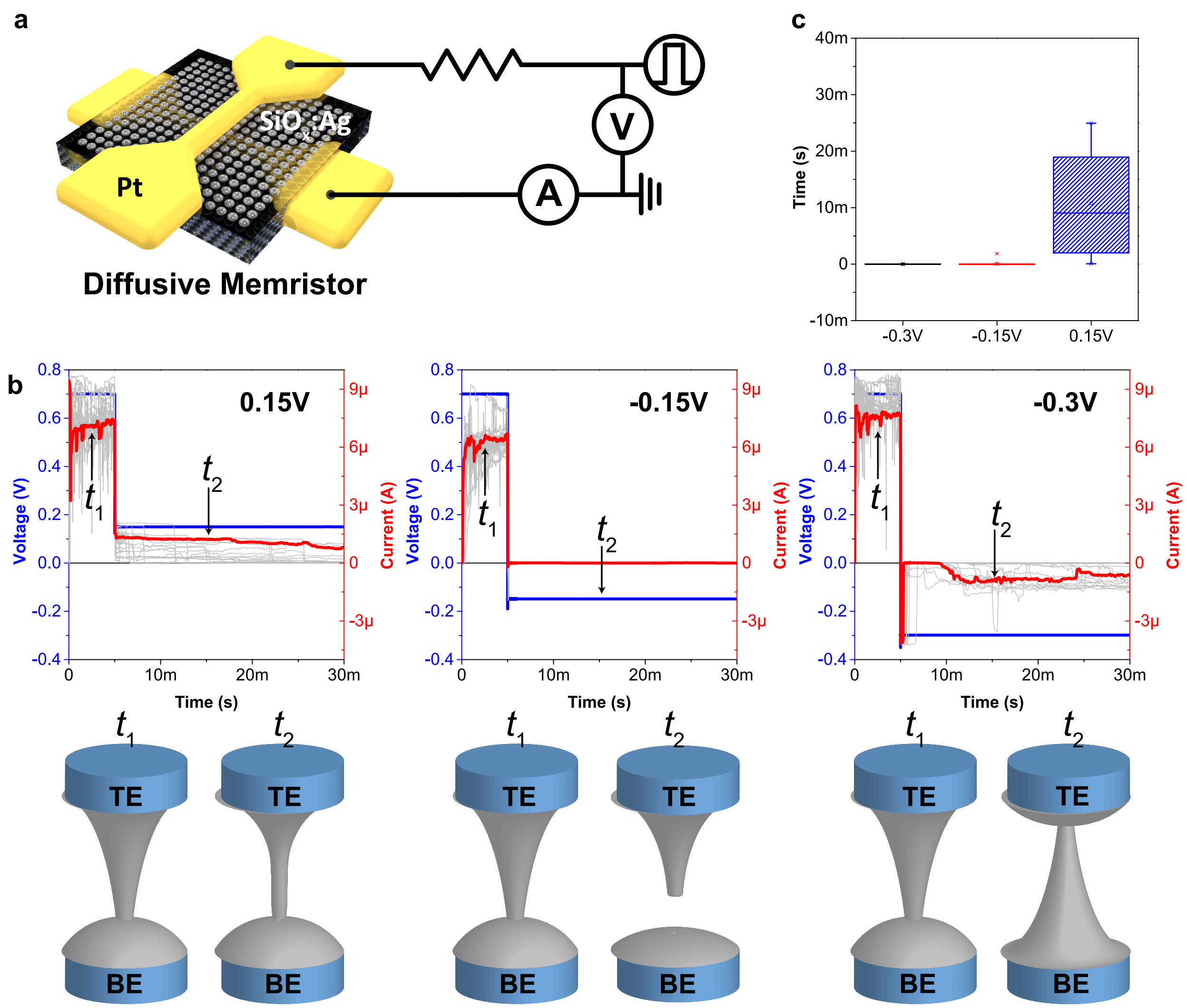

Supplementary Figure 2. Biasing dependent relaxation of a diffusive memristor. a, Schematics of the measurement setup where the diffusive memristor is in series with a current compliance resistor. **b**, The current response of a diffusive memristor to a super-threshold voltage pulse followed by different sub-threshold reading bias. Voltage and current are depicted by blue and red curves, respectively, where one typical current curve is highlighted in red. Time t_1 and t_2 are framed within the first and second phases of biasing, respectively. The device was switched ON by a positive voltage spike which drove Ag ions across the dielectric film until a stable filament had been formed. In the case a small positive sub-threshold voltage (0.15V) was applied during relaxation, the conductance relaxed slowly as the bias tended to retain the filament from the top electrode. A small negative bias (-0.15V) accelerated the filament rupture. However, a large negative bias (-0.3V) first ruptured the early filament and subsequently formed new filament with

opposite growth direction. **c**, The distribution of the observed relaxation time in **b**. The relaxation time is defined as the duration to reach 10% of its peak conductance.

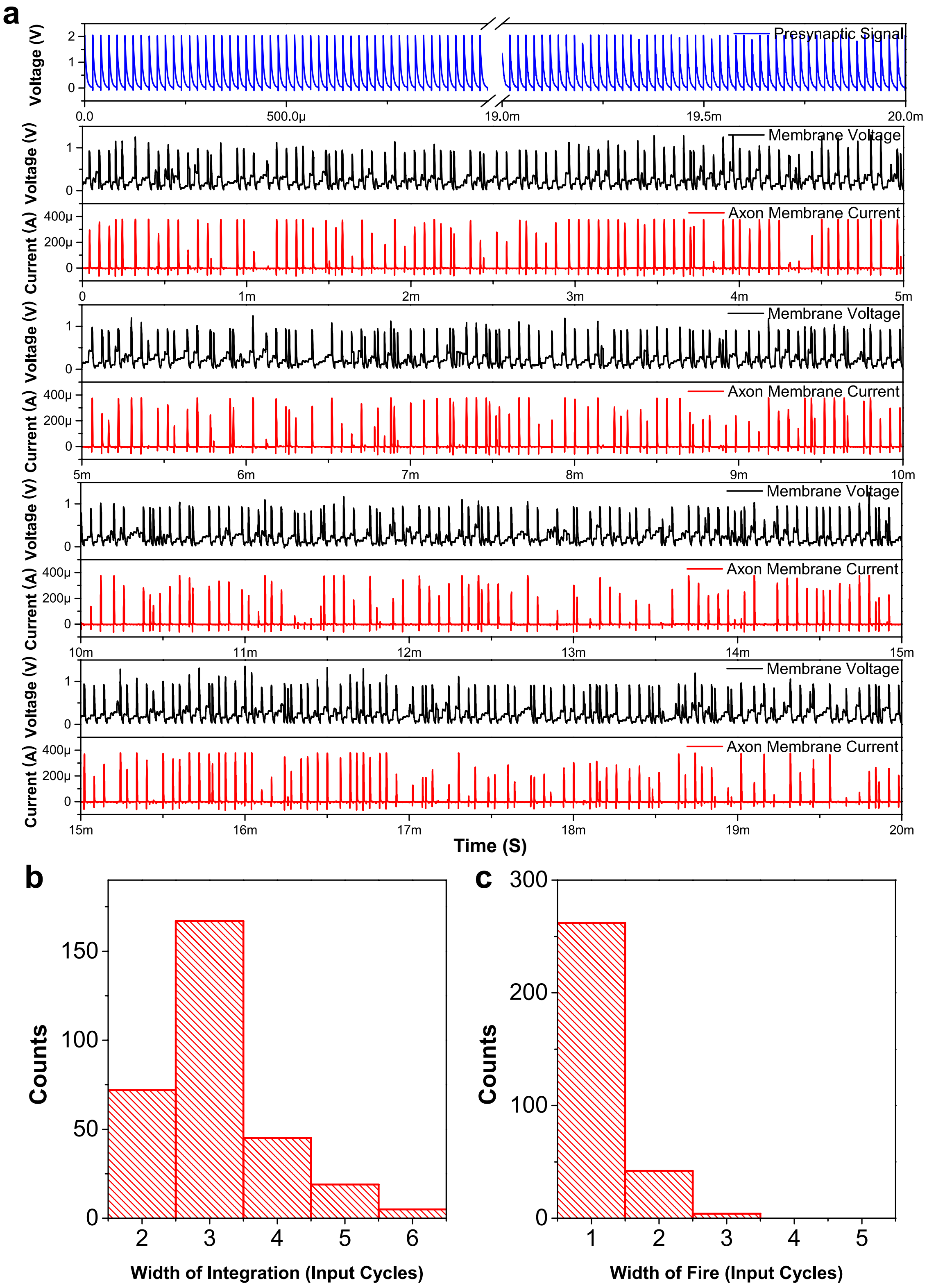

Supplementary Figure 3. Stochastic firing behavior of the neuro-transistor. a, The membrane voltage (black lines) and axon membrane current (red lines) responses to a train of 1000 pre-

synaptic voltage spikes (blue lines). **b**, The Poisson-like distribution of the width of integration in **a**. The distribution is attributed to the inherent stochasticity of the ON switching of the diffusive memristor. The width of an integration event is defined as the number of input cycles taken to rise the membrane voltage to 0.55V. **c**, The exponential-like distribution of the width of fire in **a**. The width of a fire event is defined as the number of consecutive cycles with peak membrane voltage larger than the 0.55V threshold.

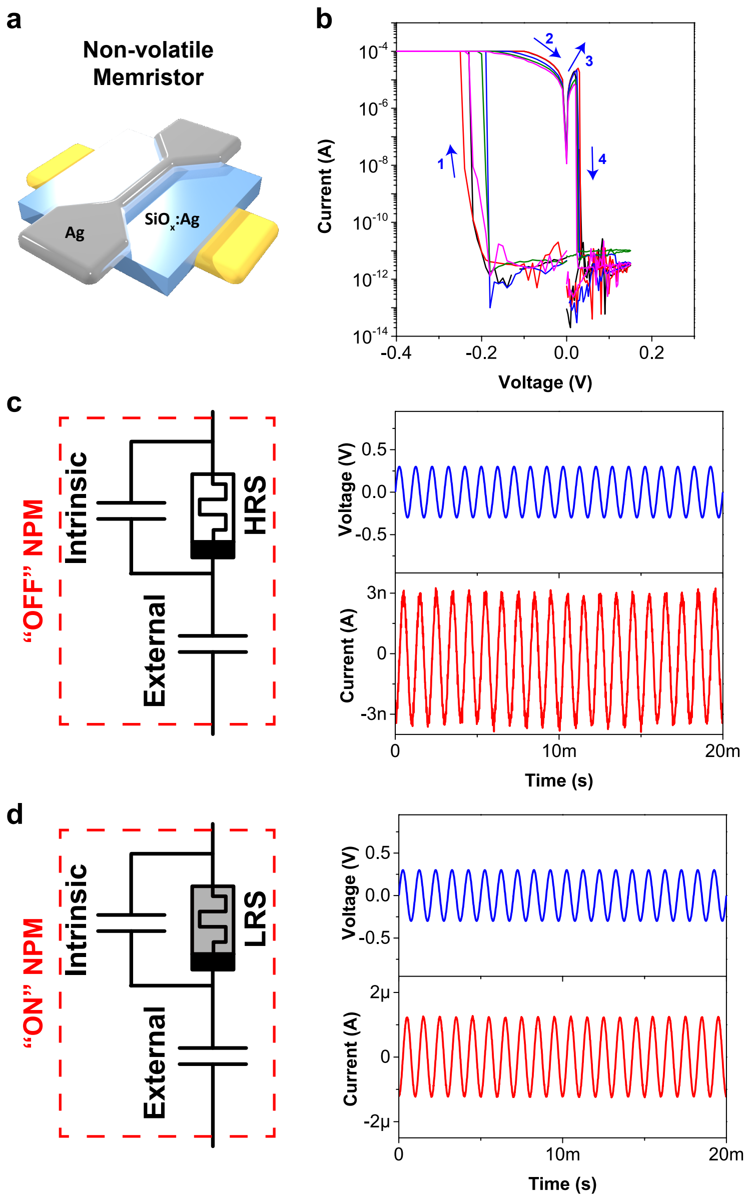

Supplementary Figure 4. Non-volatile pseudo-memcapacitor (NPM) properties. a, Schematic of the electrochemical metallization cell with SiO_x:Ag dielectrics embedded by Ag and Pt electrodes. **b**, Non-volatile bipolar resistive switching DC I-V of the electrochemical metallization cell. External current compliance of 50µA was imposed in SET process. **c-d**, Equivalent circuits and temporal current-voltage responses of the low capacitance state and high capacitance state of a NPM by wiring the electrochemical metallization cell with a 680pF capacitor, respectively. The amplitude of the sinusoidal current observed is ~3nA(~1.2µA) with a ~90 degrees phase difference from the input voltage waveforms, indicating ~2.5pF(680pF) capacitance.

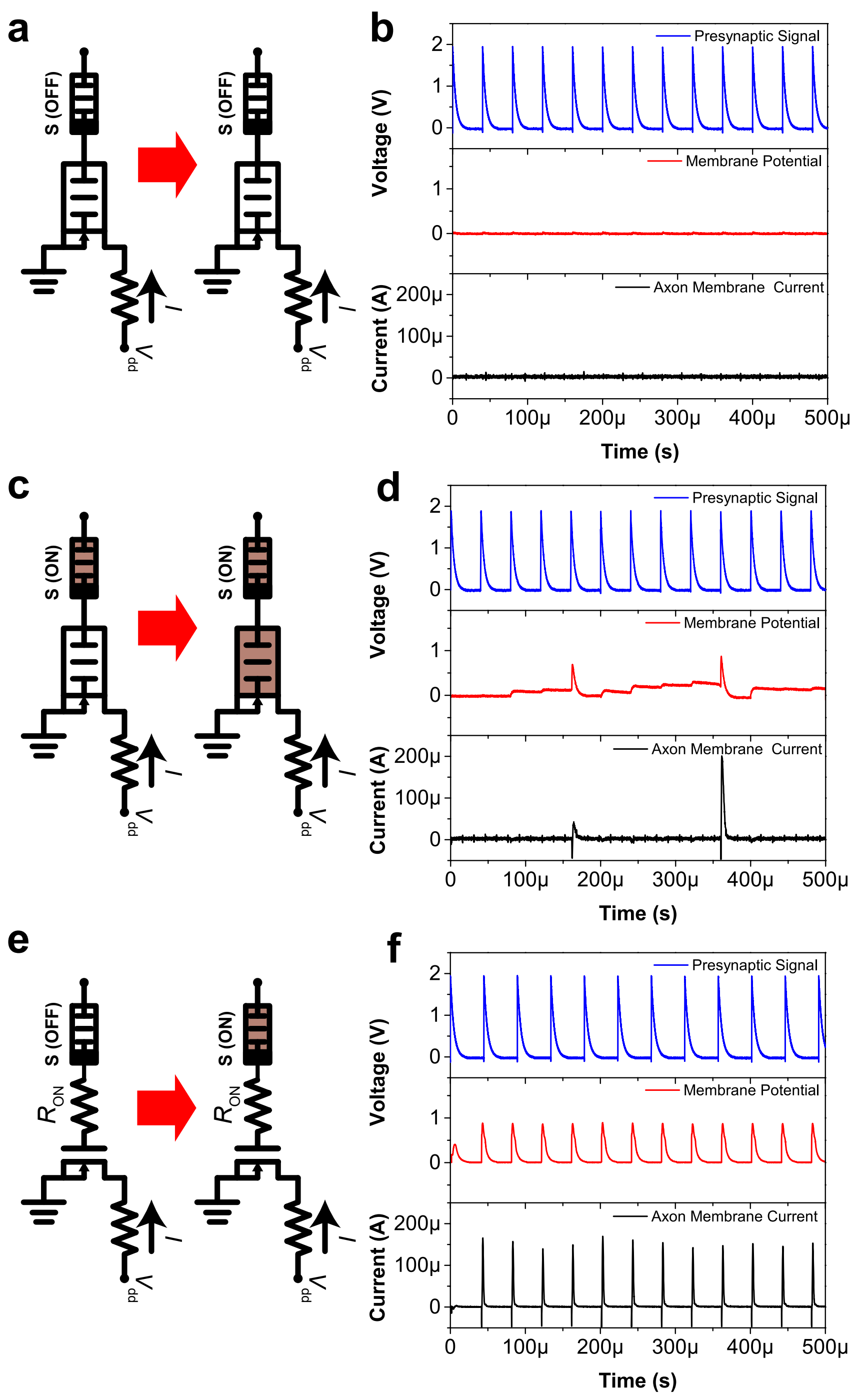

Supplementary Figure 5. The Hebbian-like synapse programming. A single synapse was connected to the post-synaptic neuron. The signal from the pre-synaptic neuron was simulated by an arbitrary waveform generator. **a**, The post-synaptic neuron could not fire together (red and black lines) with the pre-synaptic neuron (blue lines) in case the synapse was in its OFF state (low capacitance state). **b**, The post-synaptic neuron integrated and fired together with the pre-synaptic neuron in case the synapse was in its ON state (high capacitance state). **c**, An OFF state (low

capacitance state) synapse could be potentiated upon the fire of both pre-synaptic and post-synaptic neurons. Here the diffusive memristor of the post-synaptic neuron was purposely stuck-ON.

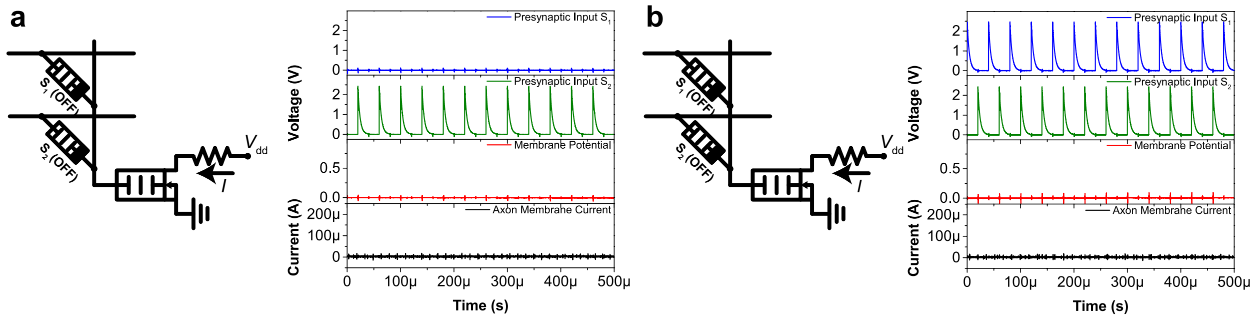

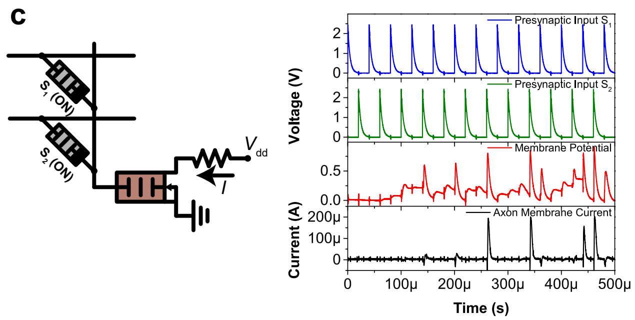

Input 1	0	2.5V	0	2.5V	2.5V	0	2.5V
Input 2	2.5V	2.5V	2.5V	0	2.5V	2.5V	2.5V
Synapse 1 (Pre)	3pF	3pF	680pF	680pF	680pF	680pF	680pF
Synapse 2 (Pre)	3pF	3pF	3pF	3pF	3pF	680pF	680pF
Neuron	Not Fire	Not Fire	Not Fire	Fire	Fire	Fire	Fire
Synapse 1 (Post)	3pF	3pF	680pF	680pF	680pF	680pF	680pF
Synapse 2 (Post)	3pF	3pF	3pF	3pF	680pF	680pF	680pF

Supplementary Figure 6. Hebbian-like learning in the capacitive neural network. a, Pre-synaptic stimulus was applied to synapse S_2 only. Both S_1 and S_2 were with low initial weights. The neuro-transistor didn't fire and none of the synapses was programmed. **b**, Pre-synaptic stimuli were applied to both synapses. The S_1 and S_2 were with low initial weights. The neuro-transistor didn't fire and none of the synapses was programmed. **c**, Pre-synaptic stimuli were applied to both synapses. The S_1 and S_2 were with high initial weights. The neuro-transistor fired. There was no programming of the synapses as both synapses were with the maximum weights. The table summarizes all possible cases of the Hebbian-like mechanism of the 2×1 capacitive neural network with binary synapses.

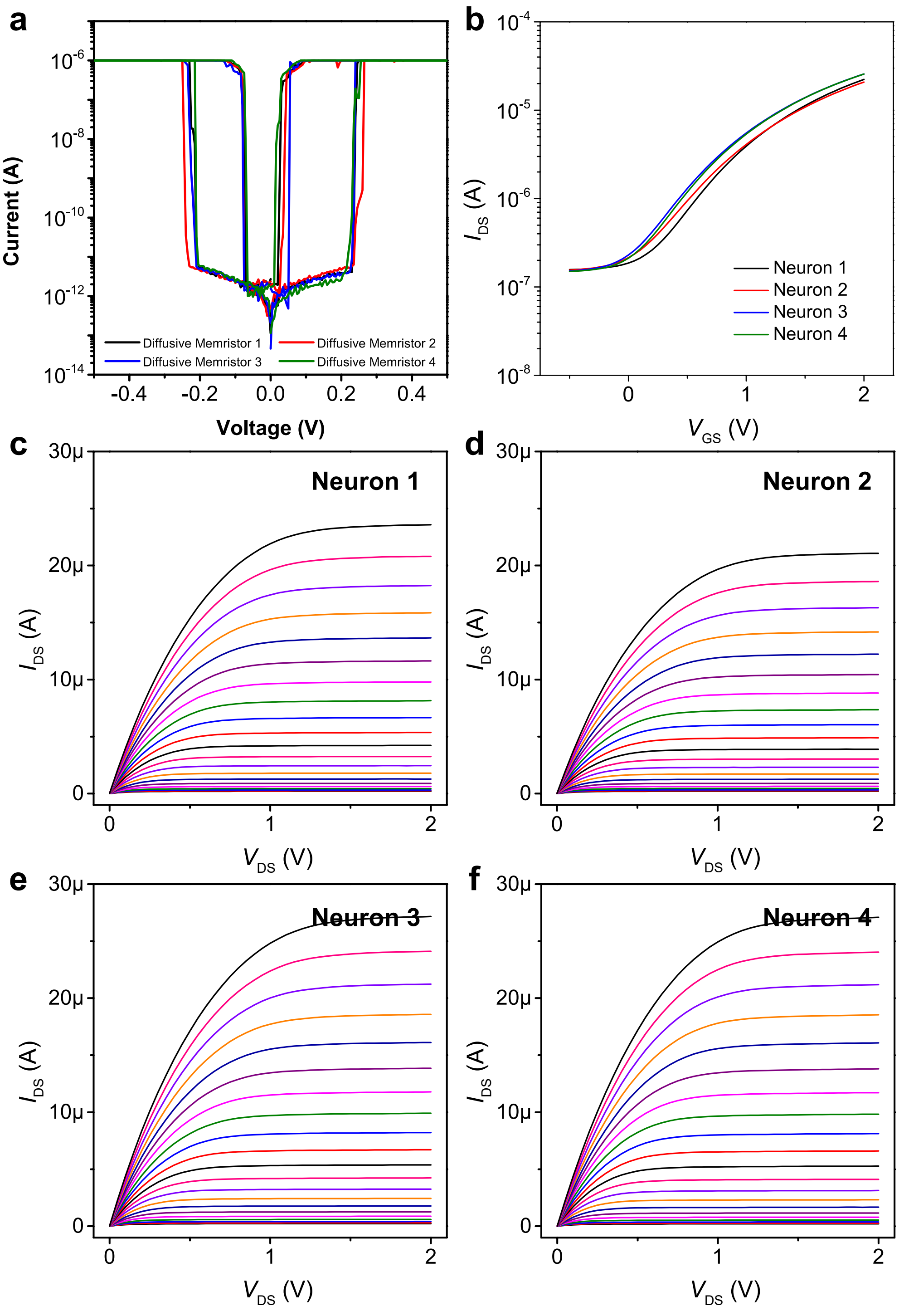

Supplementary Figure 7. Properties of individual diffusive memristors and transistors. a, DC I-V characteristics of the integrated diffusive memristor of the neuro-transistor showing threshold switching with V_{th} ~0.3V. Symmetric hysteresis loops were observed with the opposite bias polarity. **b**, Transfer characteristics (relationship between drain-source current I_{DS} and gate voltage V_{GS}) of the transistors of the integrated neurons. **c-f**, Relationship between I_{DS} and V_{DS} of

the transistors of the integrated neurons. The gate voltage V_{GS} ranges from 0.1V to 2V with a step 0.1V.

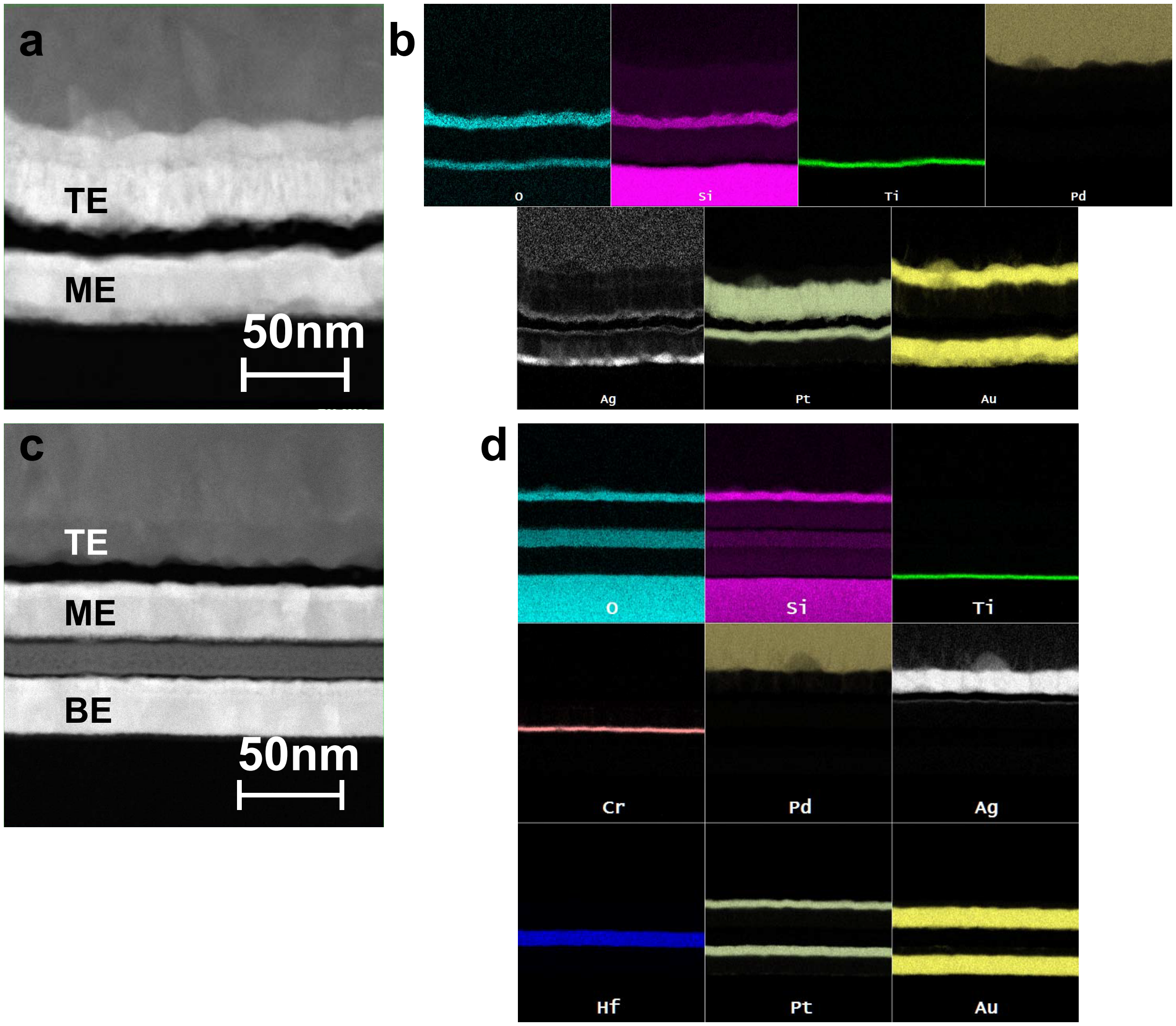

Supplementary Figure 8. Energy-dispersive X-ray spectroscopy element maps. a, The high-angle annular dark-field (HAADF) cross-sectional image of the DPM of a neuro-transistor. **b**, The corresponding element map in **a** shows thin Ag layers of the diffusive memristor. **c**, The HAADF cross-sectional image of the NPM synapse. **d**, The corresponding elemental map of **c** shows thick Ag electrodes of the electrochemical metallization cell.

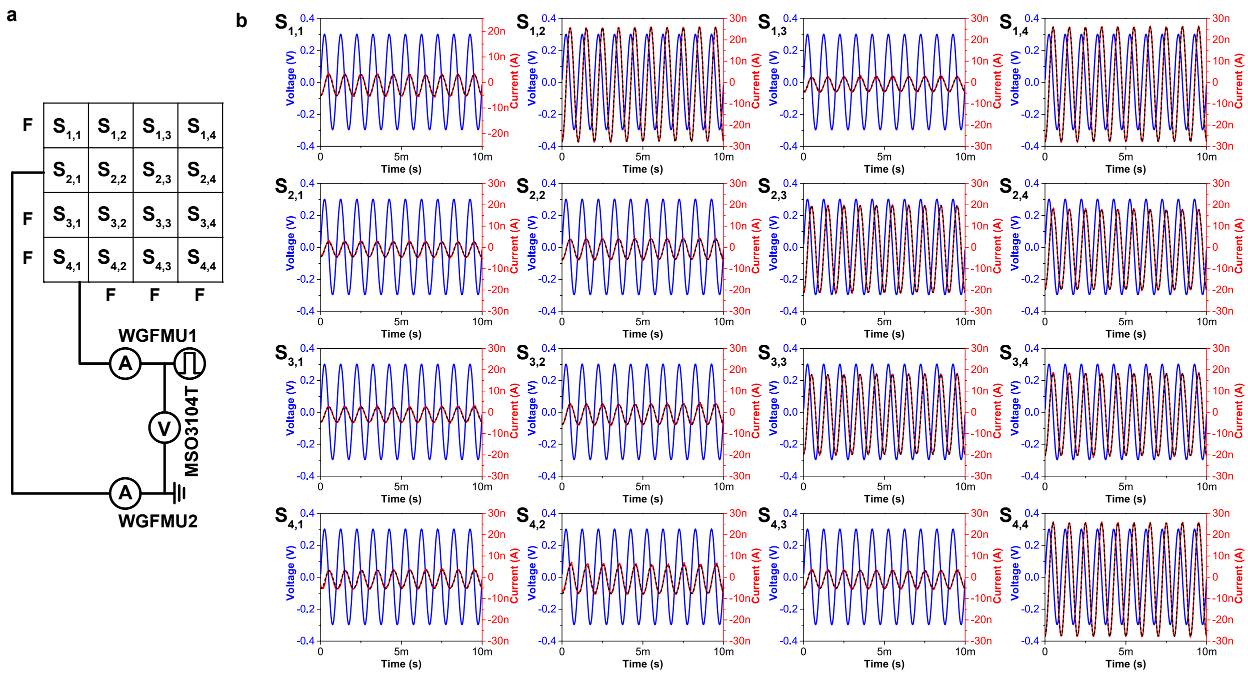

Supplementary Figure 9. Initial capacitance profile of the synaptic array in Fig. 5. a, Schematic of the measurement setup for characterizing the equivalent capacitance of individual synapse. The capacitance measurement is performed by applying a sinusoidal voltage signal to the top electrode of the synapse and measuring displacement current at the bottom plate of the capacitor part. **b**, temporal current-voltage responses of the synaptic array.

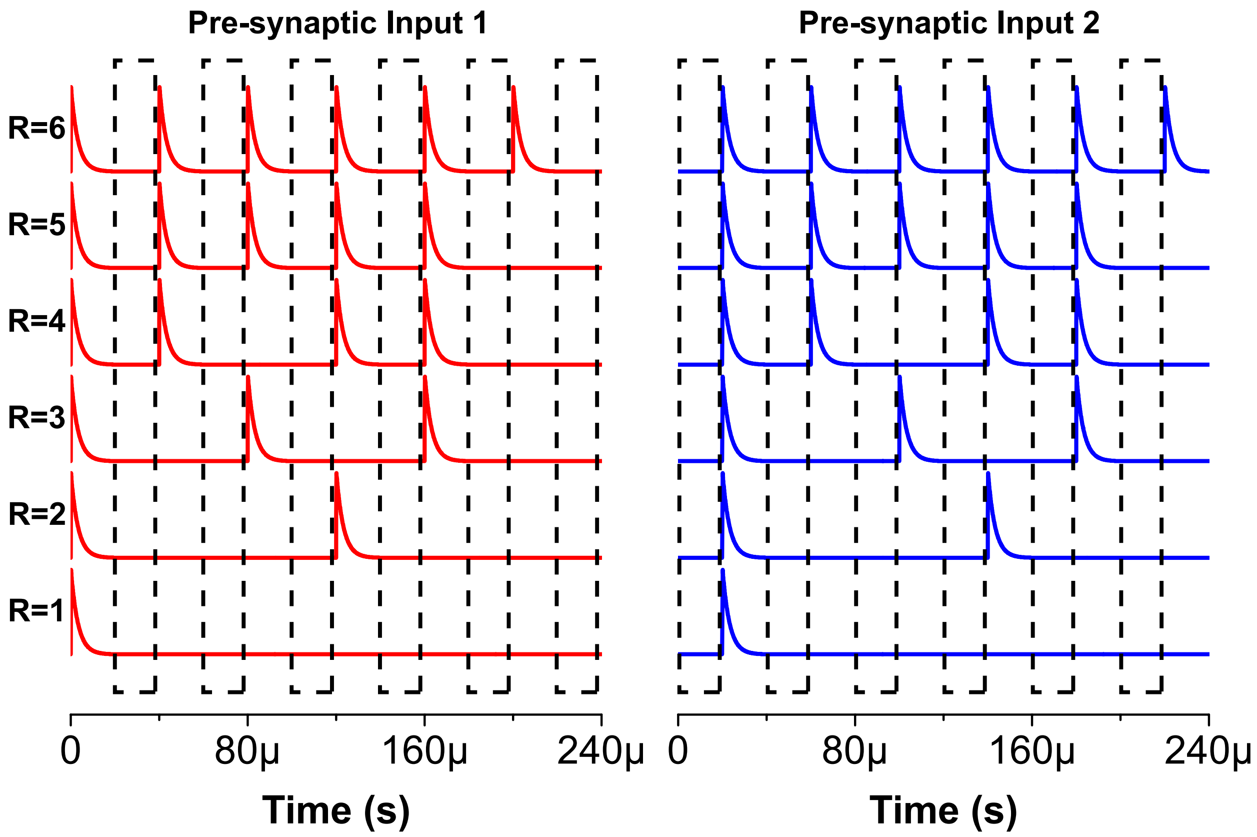

Supplementary Figure 10. The pre-synaptic inputs to the neural network in Fig. 5. Six different rates were adopted for each pre-synaptic neuron. A time-division multiplexing scheme was employed that one pre-synaptic signal would be active in the first period (e.g. 20μs) while the other input would be high-impedance or floating (black boxes). This was reversed in the next period of 20μs. As the input was only floating when the corresponding input signal was at zero potential, which maps signal "0" to high-impedance equivalently.

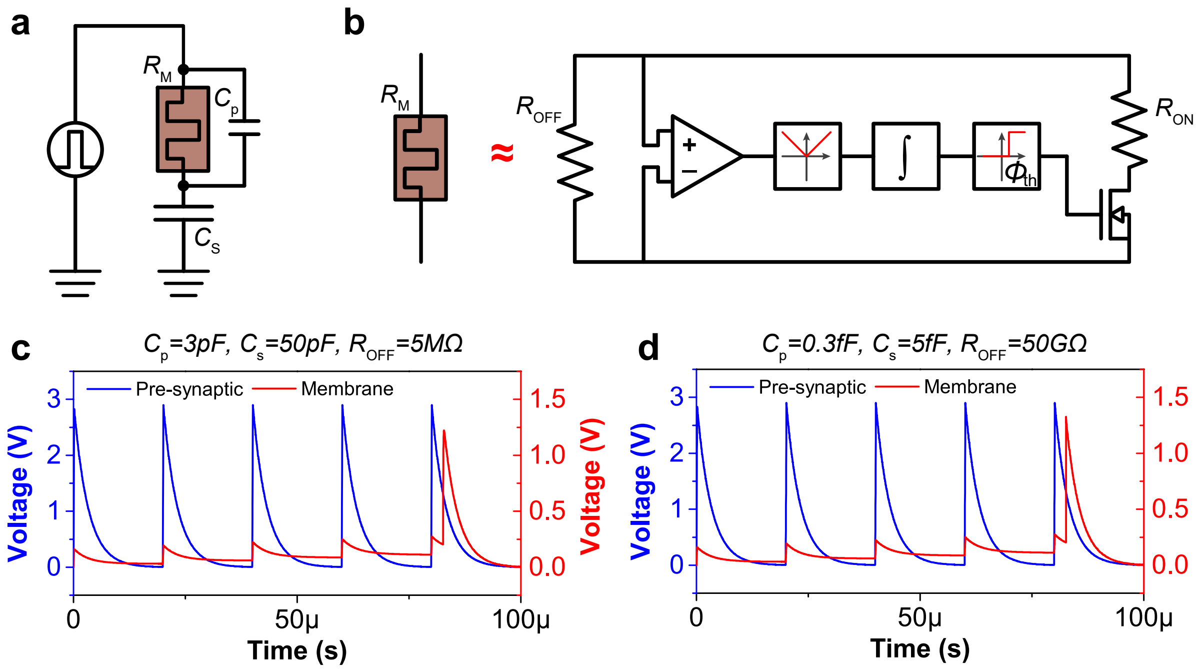

Supplementary Figure 11. Simulated scaling impact on the integrate-&-fire of a DPM. a, Circuit of the DPM scaling simulation. **b**, Equivalent block diagram of the simple diffusive memristor model. The absolute voltage across the diffusive memristor is integrated over time, representing the voltage flux which relates to the amount of Ag within the dielectrics. The device resistance switches from R_{OFF} to R_{ON} ($R_{\mathrm{ON}} \gg R_{\mathrm{OFF}}$) once the flux exceeds the threshold value $\phi_{\mathrm{th}} = 3.75 \times 10^{-5} V \cdot s$. **c-d**, Simulated membrane potential responses before and after 100× down scaling of photolithograph mask patterns, respectively. The responses are identical. Note that $R_{\mathrm{ON}} = 1\mathrm{k}\Omega$ is not scaled due to the filament nature.

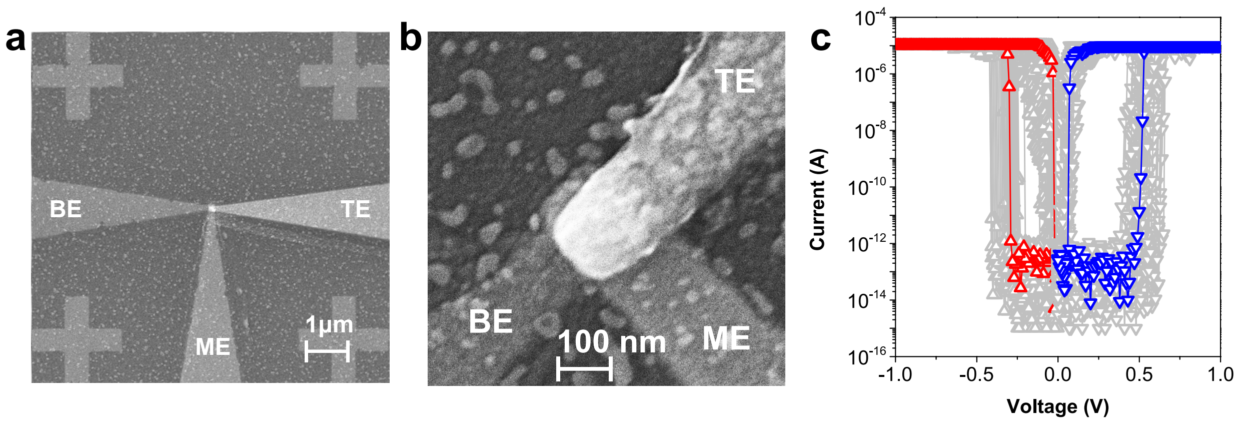

Supplementary Figure 12. Nanoscale diffusive memristor based DPM. a-b, Scanning electron microscope images of the nanoscale memcapacitor. The bottom electrode (BE) and middle electrode (ME) embed the series capacitance, while the top electrode (TE) and ME connects to the integrated diffusive memristor with ~100nm×100nm junction size. **c**, The repeatable bipolar threshold switching of the individual Pt/Ag/SiO_x:Ag/Ag/Pt diffusive memristor.

Supplementary Notes

Supplementary Note 1: Scaling impact on integration, functionality, and power of the pseudo-memcapacitive network

All elements of the pseudo-memcapacitive network chip could laterally scale with the same factor, which does not introduce any change to the network functions but reduce both chip area and operating power compared to those without scaling.

Scaling impact on integration and functionality. Here we discuss the scaling of all lateral lithography mask patterns by a factor k while keeping vertical dimensions (e.g. deposition thickness) unchanged. Such lateral scaling could be easily implemented by varying the magnification of the reduction lens in mask stepper systems. It is also worth noting that the recent launch of extreme ultraviolet lithography could potentially make the neuro-transistor gate cross-section a similar size with that of a memristor filament[1], which indicates the potential of ultrahigh density integration. The capacitance of a basic building block, a Metal-Insulator-Metal (MIM) stack, after scaling is given by Supplementary Equation 1.

$$C_{\text{after}} = \varepsilon \frac{A}{k^2 d} = \frac{1}{k^2} C_{\text{before}} \qquad (1)$$

Note that C_{after} and C_{before} denote the capacitance after and before scaling, respectively. The dielectric permittivity is ε. The lateral area of the device before scaling is A. The dielectric thickness is d. Since every element is subjected to the same scaling factor, the capacitance ratio between arbitrary two elements remains the same after scaling. It shall be noted that the scaling also applies to parasitic capacitance. Therefore, the node voltages after scaling all lithography patterns with the factor k should not change.

In addition, the integrate-&-fire behavior is retained after scaling. This is because the localized filamentary switching of diffusive memristor depends on the amount of voltage flux it has received. Here we simulate the scaling effect on the integrate-&-fire process of a single volatile pseudo-memcapacitor, as shown in Supplementary Figure 11a. A simple diffusive memristor model is used to capture the voltage flux induced ON switching, with the block diagram shown in Supplementary Figure 11b. The absolute voltage across diffusive memristor is integrated over time, representing the voltage flux experienced by the memristor. The magnitude of such voltage flux correlates to the amount of Ag within the dielectric gap from both Ag electrodes before the first

ON switching. The diffusive memristor resistance switches from R_{OFF} to R_{ON} ($R_{\mathrm{ON}} \gg R_{\mathrm{OFF}}$) once the flux exceeds the threshold value ϕ_{th}, which follows the general definition of a memristor.[2] Note this model does not cover the RESET process which is spontaneous for the volatile memristors. To prove that the integrate-&-fire behavior is invariant with scaling, we performed simulation with two sets of circuit parameters as shown in Supplementary Figure 11c-d, corresponding to that before and after 100× down scaling of photolithograph mask patterns. Please note that R_{ON} does not scale because the filamentary switching is localized. Supplementary Figure 11c shows the simulated integrate-&-fire behavior of a microscale DPM, consistent with the experimental observation shown in Fig. 1d of the main text. More importantly, it is observed that the integrate-&-fire behavior of a scaled DPM in Supplementary Figure 11d is identical with that of the microscale device, implying the integrate-&-fire behavior is scaling invariant.

We also experimentally fabricated nanoscale DPM as shown in Supplementary Figure 12a-b. Like the microscale device of Fig. 1a of the main text, it consists of 3 electrodes. The lower two electrodes embed the series capacitance while the upper two connects to the integrated diffusive memristor of ~100nm×100nm junction size. The nanoscale diffusive memristor shows consistent electrical responses under voltage sweeps with that of microscale devices, which verifies that the Ag-dynamics oriented switching of diffusive memristor is localized and scaling independent. (Please note that the built-in capacitance of the oscilloscope probes prevents electrical measurement of the "membrane potential" in the phase of integration within the nanoscale DPM.)

Scaling impact on power consumption: The capacitive crossbar network features low operating power as DPMs store electric energy in electrostatic field. Scaling all lithography mask patterns will further lower down the overall operation power because of two reasons. The first is that the scaled capacitive elements are of less energy storage capacity, thus requiring less driving power of the voltage sources or pre-synaptic neurons. On the other hand, the neuro-transistors, after scaling, could feature a smaller heat loss due to the increased resistance of pull-up resistors.

For the electrostatic fields, the energy stored in a MIM capacitive element is given by Supplementary Equation 2.

$$E_{\mathrm{after}} = \frac{1}{2} C_{\mathrm{after}} V^2 = \frac{1}{2k^2} C_{\mathrm{before}} V^2 = \frac{1}{k^2} E_{\mathrm{before}} \qquad (2)$$

The stored energy after and before scaling are denoted as E_{after} and E_{before}, respectively. The voltage across the capacitive element is V. In addition, it shall be noted that ramping up the pre-

synaptic neuron voltages charges the capacitive network, which withdraws energy from the signal sources. In contrast, ramping down the pre-synaptic neuron voltages discharges the crossbar array. Therefore, the pre-synaptic neurons receive energy from the capacitive network. At steady state, the net power is zero.

For the Joule heat loss of the pull-up resistors of the neuro-transistors (See Fig. 1e of the main text), the ON switching of the DPM neuro-transistor gates introduce current flows across the pull-up resistors which convert electricity to Joule heat. The resistance of the pull-up resistors scales according to Supplementary Equation 3.

$$R_{\mathrm{after}} = \rho \frac{k^2 d}{A} = k^2 R_{\mathrm{before}} \qquad (3)$$

The resistance after and before scaling are R_{after} and R_{before}, respectively. The resistivity of the resistive medium is ρ. The scaled power is given by Supplementary Equation 4.

$$P_{\mathrm{after}} = \frac{V^2}{R_{\mathrm{after}}} = \frac{V^2}{k^2 R_{\mathrm{before}}} = \frac{1}{k^2} P_{\mathrm{before}} \qquad (4)$$

The equation implies that the heating power P_{after} scales with the same factor as the electrical charging/discharging power. It is also much smaller than the original power P_{before}. In addition, it shall be noted that the simultaneous scaling of both capacitive and resistive elements will not affect the RC time constant for charging the next stage, which makes the response of network invariant.

Supplementary References

1 Liu, Q. *et al.* Real-time observation on dynamic growth/dissolution of conductive filaments in oxide-electrolyte-based ReRAM. *Adv. Mater.* **24**, 1844-1849, (2012).

2 Strukov, D. B., Snider, G. S., Stewart, D. R. & Williams, R. S. The missing memristor found. *Nature* **453**, 80-83, (2008).
